# Evaluation of Ketorolac Tromethamine Microspheres by Chitosan/Gelatin B Complex Coacervation

**DOI:** 10.3797/scipharm.0903-16

**Published:** 2009-12-19

**Authors:** Sanat Kumar Basu, Kunchu Kavitha, Mani Rupeshkumar

**Affiliations:** 1 Division of Pharmaceutics, Department of Pharmaceutical Technology, Jadavpur University, Kolkata – 700 032, India; 2 Department of Pharmaceutics, Bharathi College of Pharmacy, Bharathi Nagara, Mandya Dist., Karnataka – 571 422, India; 3 Department of Pharmacology, Bharathi College of Pharmacy, Bharathi Nagara, Mandya Dist., Karnataka – 571 422, India

**Keywords:** Ketorolac tromethamine, Chitosan, Gelatin B, Complex coacervation, Microspheres

## Abstract

Microspheres (MS) of Ketorolac Tromethamine (KT) for oral delivery were prepared by complex coacervation (method-1) and simple coacervation (method-2) methods without the use of chemical cross–linking agent (glutaraldehyde) to avoid the toxic reactions and other undesirable effects of the chemical cross-linking agents. Alternatively, ionotropic gelation was employed by using sodium-tripolyphosphate (Na-TPP) as cross linking agent. Chitosan and gelatin B were used as polymer and copolymer respectively. All the prepared microspheres were subjected to various physico-chemical studies, such as drug-polymer compatibility by Thin Layer Chromatography (TLC) and Fourier Transform Infra Red Spectroscopy (FTIR), surface morphology by Scanning Electron Microscopy (SEM), frequency distribution, encapsulation efficiency, in-vitro drug release characteristics and release kinetics. The physical state of drug in the microspheres was determined by Differential Scanning Calorimetry (DSC) and X-ray powder Diffractometry (XRD). TLC and FTIR studies indicated no drug-polymer incompatibility. All the MS showed release of drug by a fickian diffusion mechanism. DSC and XRD analysis indicated that the KT trapped in the microspheres existed in an amorphous or disordered-crystalline status in the polymer matrix. It is possible to design a controlled drug delivery system for the prolonged release of KT, improving therapy by possible reduction of time intervals between administrations.

## Introduction

The present study reports a novel attempt to prepare complex coacervates of chitosan and gelatin B as carriers for the widely used Non-Steroidal Anti Inflammatory Drug (NSAID) Ketorolac tromethamine.

Ketorolac tromethamine is a potent non narcotic analgesic with moderate anti-inflammatory activity. It has been investigated extensively for use in post-operative analgesia both as a sole agent and supplement opioid analgesics and excellent applicability in the emergency treatment of post operative cancer pain and in the treatment of migraine pain [1]. The biological half-life of KT is 4–6 h. Therefore frequent dosing is necessary to sustain the action of drug to alleviate pain in post operative patients with a possibility of patient non compliance. When administered as the conventional formulation, it causes gastro intestinal complications including irritation, ulcer, bleeding and perforation [2–4].

Chitosan or β-(1→4)-2-amino-2-deoxy-D-glucose is obtained by hydrolysis of the amino acetyl groups of chitin, a polysaccharide found in a wide variety of crustaceans, insects and fungi. Chitosan with excellent biodegradable and biocompatible characteristics is a hydrophilic biopolymer. Due to its unique polymeric cationic character and its gel and film forming properties, chitosan has been examined extensively used in the pharmaceutical industry for its potential in the development of drug delivery system [5–7].

The use of complexation between oppositely charged macro molecules to prepare chitosan beads or microspheres as a controlled release formulations has attracted much attention, because this process is very simple and mild [8–11]. As coacervation can be induced in systems containing both cationic and anionic hydrophilic colloids, complex coacervation is likely to occur between chitosan, a water soluble cationic polysaccharide which is soluble in dilute acids at pH 5-6, and type B gelatin, a protein which is negatively charged at pH values above its isoelectric point (pH 4.7–5.2, exactly at pH 5) [12–14].

Recently reversible physical cross-linking by electrostatic interaction, instead of chemical cross-linking is applied to avoid possible toxicity of reagents and other undesirable effects. Na-TPP is a poly anion, and can interact with cationic chitosan by electrostatic forces [15–16]. Maculotti Katia et al reported a novel formulation based on chondroitin sulphate/chitosan microspheres (CS/CH) prepared by a new emulsion-complex coacervation method for protein delivery [17]. Absorption of antigens onto chitosan microparticles via electrostatic interaction is a common and relatively mild process suitable for mucosal vaccine [18–20].

## Experimental

### Materials

Ketorolac tromethamine was obtained from (Symed Labs Limited, Hydrabad, India) as a gift sample, chitosan with a degree of deacetylation of > 85% and viscosity of 500cps at 1% (w/v) in 1 % (v/v) aqueous acetic acid (pH 5) at 20°C was supplied from (Central Institute of Fisheries and Technology, Cochin, India) as a gift sample and was used as received. Type B gelatin, bloom strength 225 received from (Sigma Chemical Company, St. Louis, USA), sodium tripolyphosphate (Na-TPP) from (Fluka Chemical Company, GmbH, Switzerland), light and heavy liquid paraffins, tween 80, acetone, glacial acetic acid, methanol and other chemicals were from (S.D. Fine Chem. Limited, Mumbai, India).

### Preparation of Ketorolac tromethamine microspheres

#### Method-1: Complex coacervation

The KT microspheres were prepared by complex coacervation technique by using chitosan/gelatin B mixture as coating material. Chitosan and gelatin were dissolved in dilute acetic acid solution (1% v/v) together at concentrations of 1–4% w/v and adjusted to a certain solution pH (usually 5.0). Ketorolac tromethamine (100mg) was dissolved in the above polymeric mixture. The drug in polymeric mixture was emulsified in 200 ml of liquid paraffin (1:1 mixture of light and heavy liquid paraffin) at 40°C containing 1 ml tween 80 (2% w/v). The emulsification time was allowed for 10min under mechanical stirring (500 rpm). The w/o emulsion was cooled to 4°C to induce coagulation of gelatin. Then 50 ml Na-TPP (1% w/v) with pH in the range 4–5 at 4°C was added drop wise. Stirring was continued for 15–60 min to obtain cross-linked microspheres. Microspheres were collected by centrifugation and washed with double distilled water several times, then with acetone to remove water and dried at room temperature under vacuum. The prepared microspheres were stored in desiccator for further studies. KT loaded microspheres with different polymer compositions (1:1, 1:2, 1:3 and 1:4) were named as K1, K2, K3 and K4 respectively.

#### Method-2: Simple coacervation

The KT microspheres were prepared by simple coacervation technique by using chitosan alone as coating material. Chitosan was dissolved in dilute acetic acid solution (1% v/v) at concentrations of 1–4% w/v and adjusted to a certain solution pH (usually 5.0). Ketorolac tromethamine (100mg) was dissolved in the above polymeric solution. The drug in polymeric solution was emulsified in 200ml of liquid paraffin (1:1 mixture of light and heavy liquid paraffin) containing 1ml between 80 (2% w/v). The emulsification time was allowed for 10 min under mechanical stirring (500 rpm). Then 50 ml Na-TPP (1% w/v) with pH in the range 4–5 was added drop wise. Stirring was continued for 15–60 min to obtain cross-linked microspheres. Microspheres were collected by centrifugation and washed with double distilled water several times, then with acetone to remove water and dried at room temperature under vacuum. The prepared microspheres were stored in desiccator for further studies. KT loaded microspheres with different polymer compositions (1:1, 1:2, 1:3 and 1:4) were named as K5, K6, K7 and K8 respectively.

### Compatibility studies

Chemical interaction between the drug and the polymeric material, if any, during the preparation of the microspheres was studied by using Thin Layer Chromatography (TLC) and Fourier Transform Infrared Spectroscopy (FTIR).

*Thin Layer Chromatography* was carried out in TLC chamber. The sample solutions of pure drug and prepared microspheres were prepared by dissolving in methanol and applied to silica gel G plates. The plates were then developed in the following solvents systems.

Solvent system 1: Dichloromethane: Acetone: Glacial acetic acid 9:1:1 (%v/v/v)
Solvent system 2: Chloroform: Methanol: Ammonia 100: 25: 1 (%v/v/v).

The R_f_ value of the pure drug as well as prepared microspheres were determined by placing the plates in an iodine chamber and the R_f_ value of pure drug was compared with the R_f_ value of prepared microspheres.

*Infrared (FTIR)* spectrum of the drug, drug loaded microspheres, blank microspheres and physical mixture of drug and empty microspheres were recorded using a FTIR (model 4100 type A, Perkin-Elmer, Norwak, CT, USA) spectrometer using KBr pellets(400–4000^−1^) with a scanning speed of 2 mm/sec with normal slit.

### Scanning Electron Microscopy (SEM)

The shape and surface morphology of the KT loaded microspheres were studied using (Jeol, JSM-840A scanning electron microscope, Japan). The gold coated (thickness 200Å; Jeol, JFC-1100E sputter coater, Japan) microspheres were subjected to secondary imaging technique at 15^0^ tilt,15mm working distance and 25 Kv accelerating voltage.

### Frequency distribution analysis

Samples of microspheres were analyzed for frequency distribution with calibrated optical microscope fitted with a stage and an ocular micrometer. Small quantities of MS were spread on a clean glass slide and the average size of 200 particles, frequency distribution was determined in each batch using the calibration factor.

### Determination of Percentage Drug Entrapment (PDE)

Efficiency of drug entrapment for each batch was calculated in terms of percentage drug entrapment (PDE) as per the following formula;

$$\text{PDE}=\frac{\text{Practical}\;\text{drug}\;\text{loading}}{\text{Theoretical}\;\text{drug}\;\text{loading}}\times 100$$

*Theoretical drug loading* was determined by calculation assuming that the entire drug present in the polymer solution used gets entrapped in microspheres, and no loss occurs at any stage of preparation of microspheres [21].

*Practical drug loading* was analyzed as follows. 20 mg of microspheres were added to 100 ml of glacial acetic acid (1% v/v) and methanol in the ratio of 3:2 and occasionally shaken for 30 min. The solution was centrifuged and 1ml of the clear supernatant was diluted to 10 ml with 0.1N HCl, the supernatant liquid was filtered through WattMann filter paper and analyzed for KT by High Performance Thin Layer Chromatography (HPTLC) [22].

### In-vitro drug release studies

Microspheres equivalent to 20mg KT were subjected to in-vitro drug release studies to assess their ability in providing the desired controlled drug delivery. Drug release studies were carried out using USP XXIII basket dissolution rate test apparatus (100 rpm, 37 ± 1°C) for 2h in1.2 pH buffer (simulated gastric fluid) and for 8h in 7.4 pH phosphate buffer (simulated intestinal fluid). At different time intervals, 5ml of the sample was withdrawn and replaced with same amount of fresh medium. The sample was analyzed for KT directly or after appropriate dilution (5–50 ml) with the pH 7.4 phosphate buffer spectro-photometrically at 320 nm using a UV/ VIS spectrometer against a reagent blank. If the absorbance (concentration) of the released drug is beyond the calibrated range of absorbance then to make it within the calibrated range the dilution of the collected dissolution sample was done by diluting with the addition of appropriate volume (5–50 ml) of dissolution medium. That dilution factor was included in the calculation of %Cumulative drug release. The changes in the surface integrity of microspheres after in-vitro drug release studies were observed by using Scanning Electron Microscope (SEM) shown in (Fig. 3h).

### Kinetics of drug release

To examine the drug release kinetics and mechanism, the cumulative release data were fitted to models representing zero-order (Q v/s t), first-order (log (Q_0_ − Q) v/s t), Higuchi’s square root of time (Q v/s t^1/2^) and Korsemeyer peppas double log plot (log Q v/s log t) respectively, where Q is the cumulative percentage of drug released at time t and (Q_0_ − Q) is the cumulative percentage of drug remaining after time t.

### Differential Scanning Colorimetry (DSC)

The physical state of drug in the microspheres was analyzed by Differential Scanning Calorimeter (Mettler-Toledo star 822^e^ system, Switzerland). The thermo grams of the samples were obtained at a scanning rate of 10°C/min conducted over a temperature range of 25–220°C, respectively.

### X-ray Diffractometry (XRD)

X-ray Diffractometry of the KT microspheres were performed by a diffractometer using model (Joel JDX-8030, Japan) equipped with a graphite crystal monochromator (Cu-Kα) radiations to observe the physical state of drug in the microspheres.

## Results and discussion

Ketorolac tromethamine MS were prepared by emulsion-phase separation technique without the use of chemical cross-linking agents: to avoid the toxic and undesirable effects such as loss of protein bioactivity, neurotoxicity of chemical cross-linking agents (usually glutaraldehyde). It can be seen that the solution pH may play an important role on the chitosan microsphere formation [16]. In this study the pH of chitosan or chitosan/gelatin mixture and the cross-linker solutions were usually adjusted to 4–5. Out of the pH region where Na-TPP interacts with chitosan, no microspheres were formed.

### Compatibility studies

**Fig. 2.**
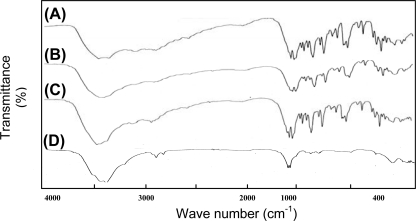
FTIR Spectrum of A) Ketorolac tromethamine B) Ketorolac tromethamine loaded microspheres C) Physical mixture of Ketorolac tromethamine and blank microspheres D) Blank microspheres.

Chemical interaction between drug and the polymeric material, if any, during the preparation of the microspheres was studied by using a TLC and FTIR. The comparable Rf values of pure drug and microencapsulated drug in the TLC study indicated the compatibility of drug with polymer and other excipients used in the preparation of KT microspheres [23]. No difference in the IR patterns of a physical mixture of the drug and blank microspheres, and drug loaded microspheres was observed (Fig. 2). Therefore, the FTIR studies ruled out the possibility of any drug polymer interaction during the preparation of microspheres [24]

### Morphological characteristics (SEM)

**Fig. 3.**
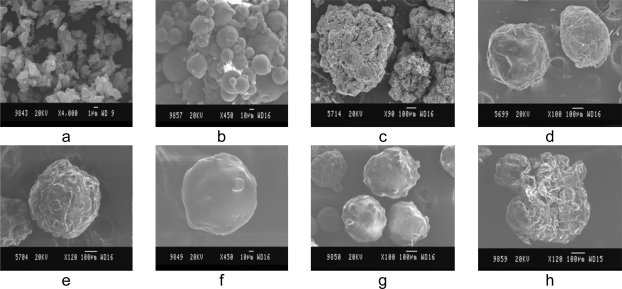
SEM Photographs of the Ketorolac tromethamine and its Microspheres. a) Ketorolac tromethamine b) Effect of stirring rate c) and d) Microspheres prepared with 1:1 and 1:4drug/polymer ratio by method 2. e) and f) Microspheres prepared with 1:1 and 1:4 drug/polymer ratio by method 1. g) Before in vitro release studies h) after in vitro release studies.

The surface morphology of the KT and KT loaded microspheres were studied by scanning electron microscopy (Fig. 3). SEM photograph of the drug indicated that the drug exists as crystals (Fig. 3a).

Surface smoothness of MS was increased by increasing the polymer concentration, which was confirmed by SEM. At lower polymer concentration (1% w/v) rough and wrinkled surface of MS was obtained (Fig. 3c and 3e) and at higher polymer concentration (4%) the MS with smooth surface was obtained (Fig. 3d and 3f).

### Frequency distribution analysis

As the drug to polymer ratio was increased, the mean particle size (MPS) of KT microspheres was also increased (Table 1). The significant increase may be because of the increase in the viscosity of the droplets (due to the increase in concentration of polymer solution). This increase is high enough to result in difficult dispersion and subdivision of droplets [25, 26].

**Fig. 4.**
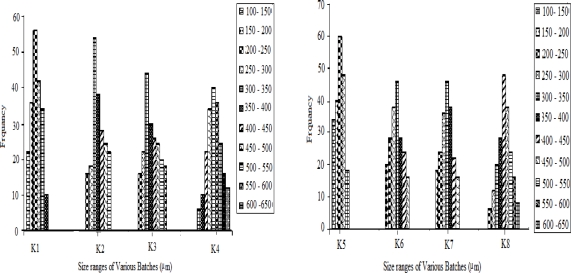
Frequency distribution of Ketorolac tromethamine microspheres.

As expected, the increase in rate of stirring decreased the mean diameter of the MS was confirmed by SEM (Fig. 3b). At 1000 rpm relatively smaller microspheres (10–100 μm) were obtained while at 500 rpm larger microspheres (100–750 μm) with normal distribution were obtained. As reported by Denkbas et al., [27] Increasing in stirring speed produces higher energy which leads to a further decrease in droplet size, thus producing smaller MS. The results of frequency distribution studies and histograms showed the normal frequency distribution of microspheres (Fig. 4).

### Drug entrapment efficiency

The drug loading efficiency of KT microspheres was determined by HPTLC method. A maximum of 37% of drug entrapment efficiency was obtained by method 1 and a maximum of 22% was obtained by method 2. It was further observed that the drug entrapment was proportional to the drug polymer ratio and size of the microspheres. By increasing the polymer concentration the entrapment efficiency was increased (Table 1).

### In-vitro drug release studies

The in-vitro release of Ketorolac tromethamine microspheres were carried out in gastric pH 1.2 for 2 h not shown in figure. The microspheres were swelled in the gastric environment but not dissolved. 10% drug was released in gastric pH at 2h. It was concluded that the 10% release also may be due to the presence of unincorporated drug on the outer surface of the microspheres which was reported in the literatures. The release study was carried out in the pH 7.4 phosphate buffer medium for 8h. It was observed that the rate of release decreased as the concentration of the carrier was increased. This may be due to low permeability of polymer to the drug.

**Fig. 5.**
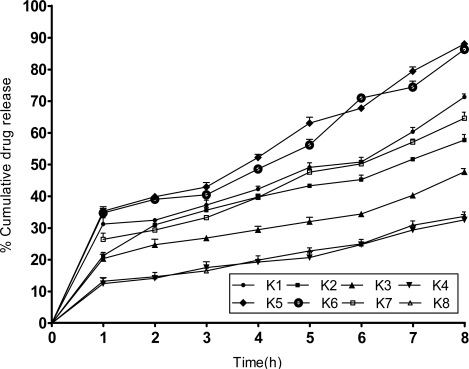
Invitro release of Ketorolac tromethamine microspheres

Slower drug release was found in the MS prepared by method 1 (maximum of 71%), compared to MS prepared by method 2 (maximum of 88%) at 8 hrs. All the parameters were run 3 times (n=3). The difference in mean of drug release of batch series ‘K’ was significant (p < 0.05). The in-vitro release profiles are shown in (Fig. 5). The data obtained were fitted to zero order, first order, Higuchi square root of time and Korsemeyer-Peppas equations to understand the mechanism of drug release from the microspheres [2]. The slopes and the regression co-efficient of determinations (r^2^) are listed in (Table 2).

The co-efficient of determination indicated that the release data was best fitted with zero order kinetics. Higuchi equation explains the diffusion controlled release mechanism. Additional evidence for the diffusion controlled mechanism was obtained by fitting the Korsmeyer-Peppas equation to the release data. The diffusion exponent ‘n’ value was found to be less than 0.5 for different drug polymer compositions, indicating fickian diffusion of drug through microspheres. All the parameters were run 3 times (n=3). The difference in mean of Zero order, First order, Higuchi kinetics and Peppas Equation between batch series ‘K’ was indicating significant (p < 0.05).

### Differential scanning calorimetry (DSC)

**Fig. 6.**
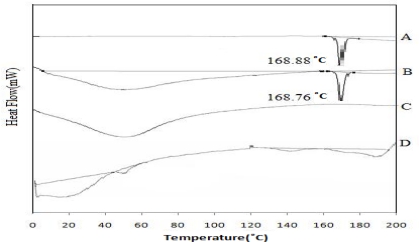
Differential scanning calorimetry thermograms A) Ketorolac tromethamine. B) Physical mixture of Ketorolac tromethamine and blank microspheres. C) Ketorolac tromethamine loaded microspheres. D) Empty microspheres.

In order to confirm the physical state of the drug in the microspheres, DSC of the drug alone, physical mixture of drug and blank micro spheres, drug loaded microspheres and blank microspheres were carried out (Fig. 6). The DSC trace of drug showed a sharp endothermic peak at 168.88°C, its melting point. The physical mixture of drug and blank microspheres showed the same thermal behavior 168.76°C as the individual component, indicating that there was no interaction between the drug and the polymer in the solid state. The absence of endothermic peak of the drug at 168.88°C in the DSC of the drug loaded microspheres suggests that the drug existed in an amorphous or disordered crystalline phase as a molecular dispersion in polymeric matrix [28–29].

### X-ray diffractometry (XRD)

In order to confirm the physical state of the drug in the microspheres, powder X-ray diffraction studies [20] of the drug alone, physical mixture of drug and blank microspheres, and drug loaded microspheres were carried out.

**Fig. 7.**
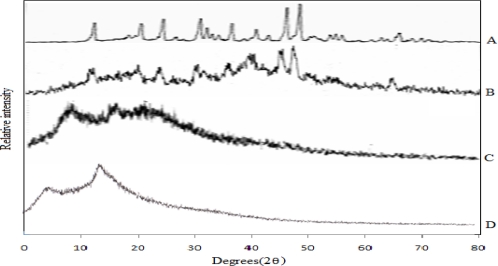
X-ray diffractograms (A) Ketorolac tromethamine. (B) Physical mixture of Ketorolac tromethamine and blank microspheres. (C) Ketorolac tromethamine loaded microspheres. (D) Empty microspheres

X-ray diffractograms (Fig. 7) of the samples described above showed that the drug is still present in its lattice structure in the physical mixture where as it is completely amorphous inside the microspheres. This may be due to the conditions used to prepare the microspheres lead to cause complete drug amorphization.

## Figures and Tables

**Fig. 1.**
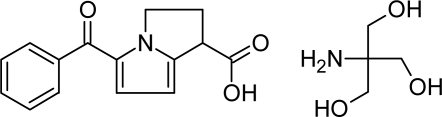
Chemical structure of Ketorolac tromethamine

**Tab. 1.** Particle size, Drug entrapment and encapsulation efficiency of Ketorolac tromethamine microspheres.

**Batch Code**	**Mean particle size(μm) mean ± SEM**	**% yield = (Practical yield / Theoretical yield) × 100**	**Drug Entrapment (%)**	**Drug Encapsulation Efficiency (%)**
K1	290.54± 6.52	90	1.33	02.39
K2	378.88± 8.73	97	2.70	07.84
K3	395.86±10.21	85	8.82	29.98
K4	542.85± 9.60	78	9.38	36.58
K5	216.84± 6.48	55	3.17	03.49
K6	314.32± 8.44	60	5.04	15.92
K7	321.06± 8.39	80	6.85	21.91
K8	432.57± 9.68	76	8.84	22.15

**Tab. 2.** Diffusion exponent (n) of Peppas model and Regression co-efficient (r^2^) of Ketorolac tromethamine release data from microspheres according to different kinetic models.

**Batch Code**	**Peppas Model (n)**	**Zero order**	**First order**	**Higuchi**
K1	0.391	0.953±0.004	0.890±0.003	0.888±0.006
K2	0.444	0.972±0.005	0.971±0.007	0.975±0.005
K3	0.373	0.952±0.006	0.923±0.003	0.899±0.008
K4	0.456	0.974±0.003	0.964±0.004	0.923±0.007
K5	0.453	0.980±0.004	0.894±0.005	0.930±0.005
K6	0.447	0.960±0.002	0.880±0.006	0.896±0.006
K7	0.447	0.987±0.007	0.962±0.002	0.946±0.004
K8	0.453	0.973±0.003	0.964±0.005	0.917±0.003

SD=Standard deviation (n=3).

The difference in mean of %Cumulative Release, Zero order, First order, Higuchi kinetics, Peppas Equation between batch series ‘K’ was significant (p < 0.05).

## References

[1] Andrade JR, Maslanka M, Maneatis T, Bynum L, Burchomore M (1994). The use of Ketorolac tromethamine in the management of post operative pain. Orthopedics.

[2] Shankar C, Mishra M (2003). Development and in-vitro evaluation of gelatin A microspheres of Ketorolac tromethamine for intranasal administration. Acta Pharm.

[3] Shyamala B, Sanmathi BS (2001). Poly(lactic acid) microspheres of Ketorolac tromethamine for parenteral controlled drug delivery system. Indian J Pharm Sci.

[4] Shyamala B, Sanmathi BS (2001). Innfluence of manufacturing parameters and the release profile of Ketorolac tromethamine from poly (Lactide-co-glycolide) microspheres. Indian Drugs.

[5] Shu XZ, Zhu KJ (2000). A novel approach to prepare tripolyphasphate/chitosan complex beads for controlled release drug delivery. Int J Pharm.

[6] Ilium L (1998). Chitosan and its use as a pharmaceutical excipient. Pharm Res.

[7] Dini E, Alexandridou S, Kiparissides C (2003). Systhesis and characterization of cross-linked chitosan microspheres for drug delivery applications. J Microencapsul.

[8] Yao KD, Peng T, Yin YJ, Xu MX, S-Rev JM (1995). Microcapsules/microspheres related to chitosan. Polymer Rev.

[9] Polk A, Amsden B, Yao KD, Peng T, Goosen MFA (1994). Controlled release of albumin from chitosan-alginate microcapsules. J Pharm Sci.

[10] Liu LS, Lius Q, Ng SY, Froix M, Ohno T, Heller J (1997). Controlled release of interleukin-2 for tumor immunotherapy using alginate/chitosan porous microspheres. J Control Rel.

[11] Peniche C, Argiieiles monal W, Peniche H, Acosla N (2003). Chitosan: An attractive biocompatible polymer for microencapsulation. J Biol Macromol.

[12] Murali Mohan Babu GV, Himasankar K, Cheruvu Narayan PS, Ramana KV (2001). Controlled release of Diclofenac sodium by gum karaya-chitosan complex coacervates: Invivo evaluation. Indian J Pharm Sci.

[13] Remunan-Lopez C, Bodmeier R (1996). Effect of formulation and process variables on the formation of chitosan-gelatin coacervates. Int J Pharm.

[14] Sinha VR, Singla AK, Wadhawan S, Kaushik R, Kumria R, Bansal K, Dhawan S (2004). Chitosan microspheres as potential carrier for drugs. Int J Pharm.

[15] Aral C, Akbuga J (1998). Alternate approach to the preparation of chitosan beads. Int J Pharm.

[16] Shu XZ, Zhu KJ (2001). Chitosan/gelatin microspheres prepared by modified emulsification and ionotropic gelation. J Microencapsul.

[17] Maculotti K, Tira EM, Sonaggere M, Perugini P, Conti B, Modena T, Pavanetto F (2009). In vitro evaluation of chondroitin sulphate-chitosan microspheres as carrier for the delivery of proteins. J Microencapsul.

[18] Xing YL, Xiang YK, Shuai S, XiuLing Z, Gang G, YuQuan W, ZhiYong Q (2008). Preparation of alginate coated chitosan microparticles for vaccine delivery. BMC Biotechnol.

[19] Gan Q, Wang T, Cochrane C, McCarron P (2005). Modulation of surface charge, particle size and morphological properties of chitosan-TPP nanoparticles intended for gene delivery. Colloids Surf B.

[20] George M, Abraham TE (2006). Polyionic hydrocolloids for the intestinal delivery of protein drugs: alginate and chitosan -a review. J Control Rel.

[21] Dubey RR, Parikh RH (2004). Two-stage optimization process for formulation of Chitosan microspheres. AAPS PharmSciTech.

[22] Gandhimathi M, Ravi TK, Shukla N (2006). Simultaneous dermination of Aspirin and Clopidogrel in tablets by HPTLC method. Indian Drugs.

[23] Manna AK, Ray S, Gupta BK, Ghosh LK (2005). Product development studies on controlled release delivery system of Nitrofurantoin. J Pharm Res.

[24] Dash AK (1997). Determination of the physical state of drug in microcapsule and micro sphere formulations. J Microencapsul.

[25] Aiedeh K, Ganasi E, Orient I, Zeeshi V (1997). Chitosan microcapsules as controlled release systems for insulin. J Microencapsul.

[26] Jeyanthi R, Mehta C, Thanoo BC, Deluca PP (1997). Effect of processing parameters on the properties of peptide-containing polylactic-co-glycolic acid (PLGA) microspheres. J Microencapsul.

[27] Denkbas ED, Seyyal M (1999). Piskins E. 5-Fluorouracil loaded Chitosan microspheres for chemo embolization. J Microencapsul.

[28] Zidan AS, Sammour OA, Hammad MA, Megrab NA, Hussain MD, Khan MA, Habib MJ (2006). Formulation of Anastrozole microparticles as biodegradable anti cancer drug carriers. AAPS PharmSciTech.

[29] Corrigan OI (1995). Thermal analysis of spray dried products. Thermochim Acta.

